# Lentivirus-mediated transgene delivery to the hippocampus reveals sub-field specific differences in expression

**DOI:** 10.1186/1471-2202-10-2

**Published:** 2009-01-13

**Authors:** Lenneke WA van Hooijdonk, Muhammad Ichwan, Thomas F Dijkmans, Theo G Schouten, Marijke WA de Backer, Roger AH Adan, Fons J Verbeek, Erno Vreugdenhil, Carlos P Fitzsimons

**Affiliations:** 1 Medical Pharmacology Department, Leiden/Amsterdam Center for Drug Research, Leiden University Medical Center, Leiden University, the Netherlands; 2 Rudolf Magnus Institute of Neuroscience, Department of Neuroscience and Pharmacology, University Medical Center Utrecht, the Netherlands; 3 Leiden Institute of Advanced Computer Science, Section Imaging & BioInformatics, Leiden University, the Netherlands

## Abstract

**Background:**

In the adult hippocampus, the granule cell layer of the dentate gyrus is a heterogeneous structure formed by neurons of different ages, morphologies and electrophysiological properties. Retroviral vectors have been extensively used to transduce cells of the granule cell layer and study their inherent properties in an intact brain environment. In addition, lentivirus-based vectors have been used to deliver transgenes to replicative and non-replicative cells as well, such as post mitotic neurons of the CNS. However, only few studies have been dedicated to address the applicability of these widespread used vectors to hippocampal cells in vivo. Therefore, the aim of this study was to extensively characterize the cell types that are effectively transduced in vivo by VSVg-pseudotyped lentivirus-based vectors in the hippocampus dentate gyrus.

**Results:**

In the present study we used Vesicular Stomatitis Virus G glycoprotein-pseudotyped lentivirual vectors to express EGFP from three different promoters in the mouse hippocampus. In contrast to lentiviral transduction of pyramidal cells in CA1, we identified sub-region specific differences in transgene expression in the granule cell layer of the dentate gyrus. Furthermore, we characterized the cell types transduced by these lentiviral vectors, showing that they target primarily neuronal progenitor cells and immature neurons present in the sub-granular zone and more immature layers of the granule cell layer.

**Conclusion:**

Our observations suggest the existence of intrinsic differences in the permissiveness to lentiviral transduction among various hippocampal cell types. In particular, we show for the first time that mature neurons of the granule cell layer do not express lentivirus-delivered transgenes, despite successful expression in other hippocampal cell types. Therefore, amongst hippocampal granule cells, only adult-generated neurons are target for lentivirus-mediated transgene delivery. These properties make lentiviral vectors excellent systems for overexpression or knockdown of genes in neuronal progenitor cells, immature neurons and adult-generated neurons of the mouse hippocampus in vivo.

## Background

The hippocampus is a brain structure that forms part of the limbic system and is involved in memory formation and spatial navigation. The *Dentate Gyrus *(DG) field, despite of being composed mainly by granule cells, is an heterogeneous structure [1]. Moreover, the subgranular zone (SGZ) of the DG, along with few other few areas of the adult brain, is characterized by the existence of ongoing neuronal generation known as adult neurogenesis [2,3]. All in all, these and other important observations have called for extensive attention to the study of the adult DG and its functions.

In this respect, one challenging task is to identify and employ genes and molecular mechanisms directly involved in hippocampal functions, such as neuronal plasticity and neurogenesis [4,5]. The ability to manipulate the genotype in vivo provides major opportunities for studying gene function in the mammalian nervous system and for developing novel therapeutic strategies [6].

Viral-mediated single-cell gene manipulation has proven to be one of the most successful approaches to study molecular mechanisms involved in adult neurogenesis in an intact brain environment, [7,8]. With this aim, retroviral vectors have been extensively used in the study of neurogenesis due to their ability to transduce only replicative cells [7,9]. Also, lentiviral vectors have been extensively used to deliver transgenes to replicative and non-replicative cells, such as post-mitotic neurons of the CNS [10,11]. Among lentiviral vectors, Vesicular Stomatitis Virus G glycoprotein (VSV-G)-pseudotyped are the most widely used due to their very broad tropism and stability of the resulting pseudotypes. Moreover, they have received considerable attention since they have recently entered human clinical applications [11]. Interestingly, numerous reports have described on the use of lentiviral vectors on hippocampal neurons in vivo [6,12-18].

Aiming to demonstrate the usefulness of modified lentiviral vectors to deliver transgenes to the adult mouse hippocampus and extensively characterize the cell types that are effectively transduced in vivo, we used a previously described VSV-G-pseudotyped advanced generation lentiviral vector (AGLV) to express the enhanced green fluorescent protein (EGFP) under the control of the cytomegalovirus (CMV) promoter [19].

EGFP expression was analyzed one and five weeks after stereotaxic injection to the mouse hippocampus and the local distribution of EGFP+ cells within different hippocampal sub-fields was compared. We identified the different cell types transduced in the DG using cell-lineage specific markers [20,21]. The distribution and location of EGFP+ cells were also analyzed and quantified in the DG and *Cornu Ammonis 1 *(CA1) fields for comparison.

We report that lentivirus-mediated transgene expression in the DG is restricted to a subpopulation of NPC and immature neurons present in the inner granule cell layer (GCL), while presumably more mature granule cells located in the outer layers are resistant to transgene expression.

These results reveal for the first time the existence of hippocampus sub-field and cell-type specific differences in lentivirus-mediated transgene expression. These properties make lentiviral vectors excellent delivery systems for studies aiming to characterize the functions of hippocampal NPC and immature neurons, where in vivo gene manipulation is requested.

## Results

### Lentivirus-mediated EGFP delivery to the DG

**Figure 1:**
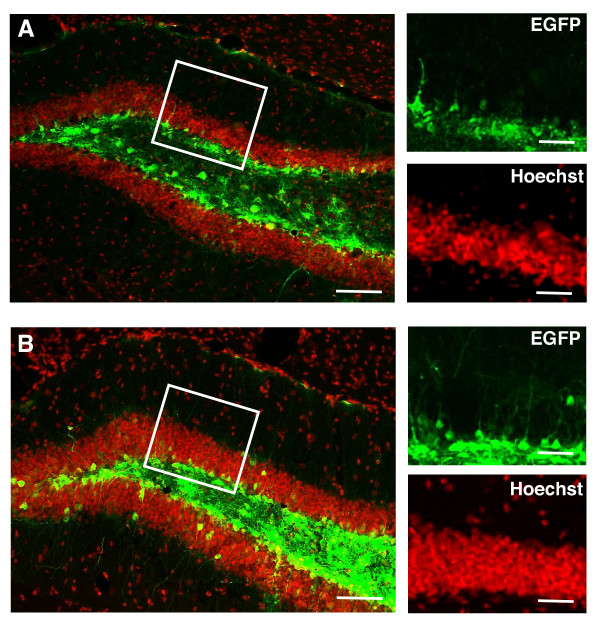
**Lentivirus-mediated EGFP delivery to the DG**. Lentivirus-mediated delivery by stereotaxic injections to the hilar region of the hippocampus does not results in substantial EGFP expression in the GCL, despite low (A) or high (B) EGFP expression, 1 week after injection. Each image shown is representative of 5 animals independently injected. Right panels represent the boxed area in the left panels of the figure. Scale bars: left panels 100 μm; right panels 20 μm.

In order to transduce cells present in the DG of the mouse hippocampus, we used a previously described AGLV system where the CMV promoter controls EGFP expression [19], further referred here as CMV-EGFP. This vector was infused by stereotaxic injection into the DG (Fig. 1). Under these experimental conditions we observed a marked restriction of EGFP expression to the hilar region and the SGZ and only few EGFP+ cells present in the GCL one week after injection (Fig. 1A). This spatial distribution is reminiscent of previous observations with murine Maloney Leukemia virus (MMLV)-derived retroviruses, transducing only dividing cells [9,22]. Notably, increased EGFP expression from higher lentiviral vector delivery titers did not result in a substantially increased proportion of EGFP+ cells located in the GCL, while the total numbers of EGFP+ cells were drastically increased, resulting in massive EGFP expression in the hilar region and the SGZ (Fig. 1B).

### Spatial distribution of EGFP+ cells in the GCL after CMV-EGFP injection

**Figure 2:**
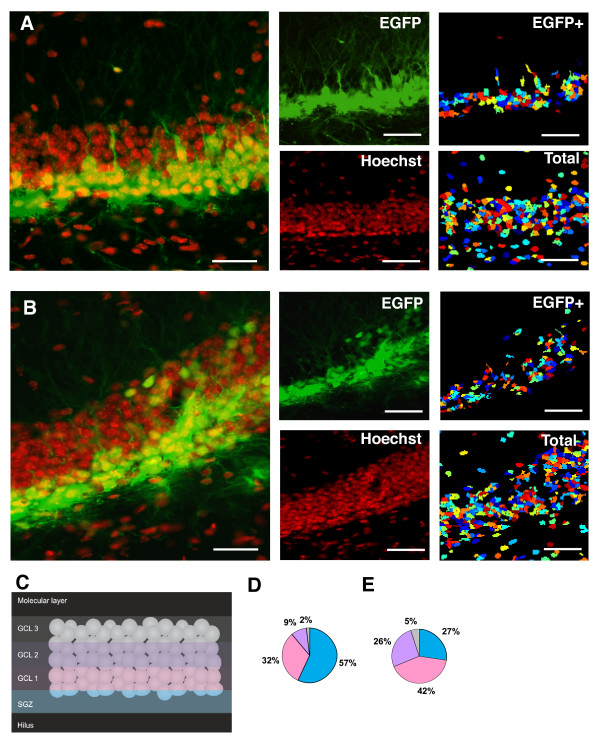
**EGFP+ cell location after injection with CMV-EGFP in the DG**. Distribution of EGFP+ cells in the GCL 1 (A) or 5 (B) weeks after stereotaxic injection of CMV-EGFP. The central panels represent the split confocal channels shown merged in left panels. Right panels depict pseudo-colored cell-localization maps, used for quantitative image analyses, generated with Cell Profiler showing the automatically identified EGFP+ and total cells. Scale bars: 20 μm. Each image shown is representative of 5 animals independently injected. C, Schematic diagram depicting the subdivisions of the GCL used for quantitative image analyses, reproduced from [24], with permission from the authors. Distribution of EGFP+ cells within the GCL 1 (D) or 5 (E) weeks after the stereotaxic injection, normalized to the total number of EGFP+ cells. Each portion of the pie diagrams represents the mean percentage of EGFP+ within internal subdivisions shown in C, indicating the distribution across the GCL, color-coded according to (C).

In order to account for the spatial distribution of the EGFP+ cells in the GCL of the DG, we subdivided the GCL in 4 different two-nucleus-wide regions, following the method described by Kempermann *et al.*, and extensively used by others [8,23,24] (Fig. 2). These four regions were designated SGZ, GCL1, 2 and 3 (Fig. 2C). Thereafter, we applied semi-automated, software assisted, quantification of the percentage of total EGFP+ cells present in each of these regions. Although the numbers of total EGFP+ were variable among different injections, as described for MMLV-based retroviral vectors [9], the relative percentages of cells present in the different subdivisions of the DG were consistently reproducible. We found that one week after stereotaxic injection, a large percentage of the cells reside in the SGZ (57 ± 1%, n = 5 animals) and the innermost layer of the GCL, GCL1 (32 ± 2%, n = 5 animals; Figure 2A, C and 2D). When the number of EGFP+ cells was assessed five weeks after stereotaxic injection, we found that the larger percentage of EGFP+ cells still resided in the SGZ (27 ± 4%, n = 5 animals) and the GCL1 (42 ± 3%, n = 5 animals) with a significantly higher percentage of EGFP+ cells located into the intermediate third of the GCL (GCL2, 26 ± 3 vs. 9 ± 4%, 5 and 1 weeks respectively, p < 0.05 Student t test, n = 5 animals each; Fig. 2B and 2E). Notably, EGFP+ cells rarely reached the outer third of the GCL (GCL3) and the percentage of cells located in the GCL3 was not significantly different from the one observed one week after injection (5 ± 3 vs. 2 ± 2%, 5 and 1 weeks respectively, n = 5 animals each; Fig. 2D and 2E).

### Spatial distribution of EGFP+ cells in the GCL after CaMKII-EGFP injection

**Figure 3:**
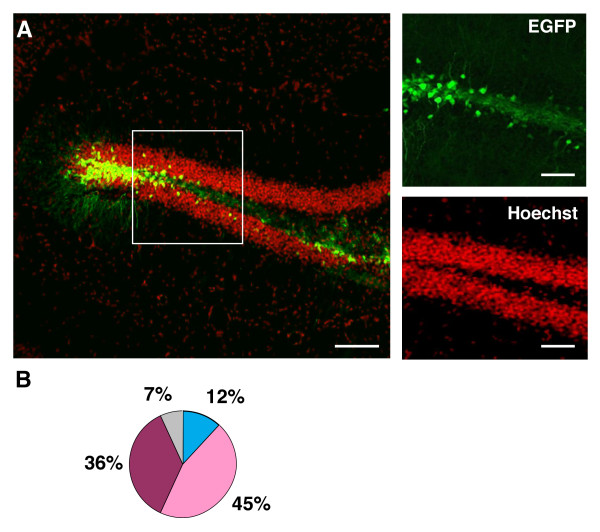
**EGFP+ cell location after injection with CamKII-EGFP in the DG**. A: Distribution of EGFP+ cells in the GCL, 1 week after stereotaxic injection with CamKII-EGFP. Right panels represent the boxed area in the left panel of the figure. Scale bars: left panel 100 μm; right panels 20 μm. Each image shown is representative of 5 animals independently injected. B: Distribution of EGFP+ cells within the GCL 1 week after the stereotaxic injection, normalized to the total number of EGFP+ cells. Each portion of the pie diagram represents the mean percentage of EGFP+ within internal subdivisions of the GCL, color-coded according to (2C).

In order to asses the possibility that the distribution of EGFP+ cells in the GCL after lentivirus transduction may depend on the promoter used to control EGFP expression, we used two other previously described lentiviral vectors where EGFP expression is controlled by neuron-specific promoters, the Synapsin I (denoted here Syn-EGFP) and the CamKII (denoted here CaMKII-EGFP) promoters. These vectors promote different levels of EGFP expression in mature post-mitotic cortical neurons, presumably due promoter's specificity for different neuronal developmental stages [10]. All lentiviral constructs were produced with the same packaging system and pseudotyped with VSV-G protein to avoid possible differences in cell-type targeting due to the use of different pseudotyping proteins [25]. When the spatial distribution of EGFP+ cells was assessed one week after CaMKII-EGFP injection, we observed that this distribution was significantly different from that observed one week after CMV-EGFP injection (Fig. 3). Injection of CaMKII-EGFP resulted in a significantly smaller percentage of EGFP+ cells present in the SGZ (12 ± 2 vs. 57 ± 1%, CaMKII-EGFP and CMV-EGFP respectively, p < 0.05 Student t test, n = 5 animals each) and a concomitant larger percentage present in GCL1 (45 ± 4%, n = 5 animals) and GCL2 (36 ± 3%, n = 5 animals). Nevertheless, GCL3 was still the layer with fewer cells, with only 7 ± 2% of the EGFP+ cells present in this particular layer (Fig. 3A–B).

These results suggested that, although the promoter used to control EGFP expression is relevant to obtain cell type specific (neuronal) expression, the outer neuronal layer of the GCL (GCL3) is not easily transduced by (VSV-G pseudotyped) lentiviral vectors.

To test this hypothesis, we utilized a lentiviral vector where the expression of EGFP was controlled by the Synapsin promoter (Syn-EGFP). This promoter has been shown to drive EGFP expression in earlier, presumably more immature, stages during neuronal development [10]. EGFP expression controlled by the Synapsin I promoter led to a pattern of distribution of EGFP+ cells in the GCL very similar to that obtained with CMV-EGFP, confirming that the promoter controlling EGFP is of relevance for the spatial distribution of EGFP+ cells in the GCL (Table 1). Nevertheless, a very small percentage of the EGFP+ was found to be in the GCL3, as observed with the other lentiviral vectors used in this study (Table 1).

**Table 1:** Distribution of EGFP+ cells in the DG of animals transduced with three different lentivirus-based vectors.

	CMV-EGFP	Syn-EGFP	CaMKII-EGFP
SGZ	57 ± 1%	45 ± 1%	12 ± 3%
GCL1	32 ± 2%	41 ± 1%	45 ± 4%
GCL2	9 ± 1%	12 ± 2%	36 ± 2%
GCL3	2 ± 1%	2 ± 1%	7 ± 2%*

Distribution of EGFP+ cells, expressed as percentage of total EGFP+ cells, with their soma within different domains of the DG (as defined in materials and methods) at 1 week post infection. Values represent mean + SEM (*n *= 5 animals; *: significantly different vs. CMV-EGFP; p < 0.05, Student t test).

### Spatial distribution of EGFP+ cells in the CA1 after CMV-EGFP injection

**Figure 4:**
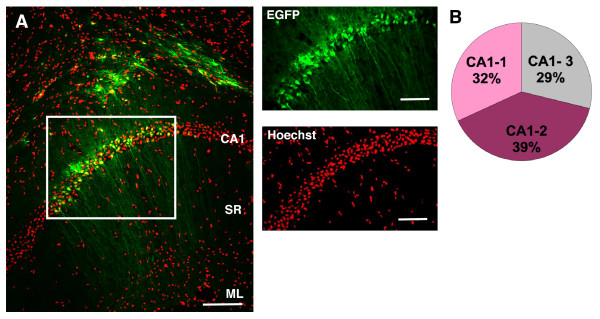
**EGFP+ cell location in CA1 after injection with CMV-EGFP in the SR**. A: Distribution of EGFP+ cells in CA1, 1 week after stereotaxic injection of CMV-EGFP to the SR. Right panels represent the boxed area in the left panel of the figure. Scale bars: left panel 100 μm; right panels 20 μm. Each image shown is representative of 5 animals independently injected. B: Distribution of EGFP+ cells within the CA1, 1 week after the stereotaxic injection, normalized to the total number of EGFP+ cells. Each portion of the pie diagram represents the mean percentage of EGFP+ within each internal subdivision of the CA1. SR: *Stratum Radiatum*; CA1: *Cornu Ammonis *1; ML: *Molecular layer*; DG: *Dentate Gyrus*.

These observations prompted us to speculate that the CMV promoter may not be highly expressed in mature neurons. To test this hypothesis we delivered CMV-EGFP to the CA1 region of the adult mouse hippocampus. One week after virus injection, we observed a strong expression of EGFP+ in the CA1 field. Typically, EGFP+ cells presented their somata in the CA1 region and extended long dendrites into the *Stratum radiatum *(SR), phenotypically resembling CA1 pyramidal cells (Fig. 4A). These results confirmed that the CMV is capable of driving EGFP expression in mature post-mitotic neurons, as previously shown by others [26].

In analogy to the procedure applied for the GCL, we arbitrarily subdivided the CA1 layer in three identical width regions (CA1-1, CA1-2 and CA1-3) and accounted the distribution of EGFP+ cells in them. EGFP+ cells were homogeneously distributed across the CA1 layer of pyramidal neurons (Fig. 4B), indicating that the irregular distribution of EGFP+ cells observed in the GCL of the DG reflects an inherent difference among granule cells in their permissiveness for lentivirus transduction.

**Figure 5:**
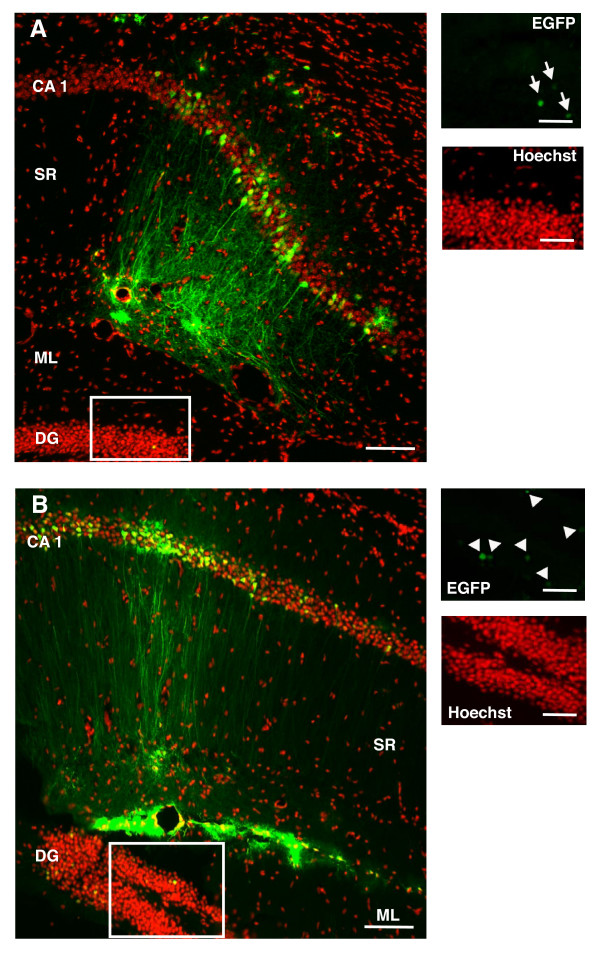
**Lentivirus-mediated EGFP delivery to the SR**. Lentivirus-mediated delivery by stereotaxic injections to the SR of the hippocampus does not result in substantial EGFP expression in the GCL, despite low (A) or high (B) EGFP expression, 1 week after injection. Right panels represent the boxed area in the left panels of the figure. Arrows (A) and arrowheads (B) indicate EGFP+ cells in the GCL. Scale bars: left panels 100 μm; right panels 20 μm. Each image shown is representative of 5 animals independently injected. SR: *Stratum Radiatum*; CA1: *Cornu Ammonis *1; ML: *Molecular layer*; DG: *Dentate Gyrus*.

To challenge this hypothesis we directed the stereotaxic injection to the SR of the hippocampus (Fig. 5), arguing that by doing so granule cells present in the outer layers (GCL3) will be directly exposed to the lentivirus, bypassing any possible physical barrier that may obstruct the free diffusion of the lentiviral suspension through the GCL when injected into the hilus. If the CMV-EGFP lentivirus would be able to transduce granule cells present in the outer shell of the DG, we should observe EGFP+ cells in the GCL3. Interestingly, we observed strong EGFP expression in cells present in the *Molecular Layer *(ML) and CA1 and even some EGFP+ positive cells in the GCL2 but none in the GCL3 (Fig. 5A). Increased EGFP expression from higher lentiviral vector delivery titers did not result in a substantially increased proportion of EGFP+ cells located in the GCL, while the total numbers of EGFP+ cells were drastically increased (Fig. 5B). These observations strengthened our conclusion that cells present in the GCL3 have inherent properties that make them less permissive to lentivirus-delivered transgene expression.

### Transduction pattern of the CMV-EGFP lentivirus vector in the DG

**Figure 6:**
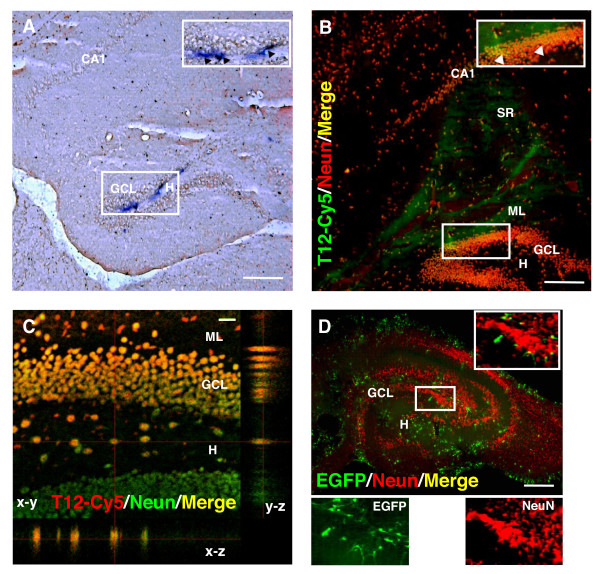
**Transduction pattern of the CMV-EGFP lentivirus vector in the DG after delivery to the SR**. A: In situ hybridization for EGFP mRNA in the GCL, 1 week after stereotaxic injection of CMV-EGFP to the SR. Inset: higher magnification view of the boxed area. Arrowheads indicate positive EGFP expression in the hilus and SGZ. B: Stereotaxic injection to the SR of a fluorescently labeled transferrin-derived peptide (T12-Cy5, pseudocolored green). NeuN+ cells are shown in red. Inset: higher magnification view of the boxed area. Arrowheads indicate NeuN+ cells in the outer GCL positively transduced with the fluorescent peptide (yellow). Animals were sacrificed 48 h after the injection. C: Higher magnification confocal image showing colocalization (yellow) of T12-Cy5 (red) and NeuN (green) in cells located across the suprapyramydal blade (top) hilus and infrapyramidal blade (bottom) of the DG. The orthogonal projection on the y-z axis shows a gradient of peptide expression from the ML to the H with highest expression in cells located in the outer GCL. D: CMV-EGFP transduction pattern in DIV-5 organotypic hippocampal slice cultures. Inset: higher magnification of the boxed area. The split panels at the bottom show the corresponding EGFP and NeuN signals from the same area. Note the almost complete lack of colocalization. Each image shown is representative of 5 animals independently injected. Scale bars: A, B and D: 100 μm; C: 10 μm. CA1: *Cornu Ammonis *1, SR: *Stratum Radiatum*; ML: *Molecular layer*; GCL: *Granule Cell Layer*;H:*Hilus*; SGZ: *Subgranular Zone*.

In order to verify our hypothesis that the lack of transduction of GCL3 neurons is a consequence of inherent cellular properties and not of technical limitations of our delivery strategy we performed a series of experiments, presented collectively in Fig. 6.

Correlational studies have demonstrated a large degree of discrepancy among transcript (mRNA) and protein expression levels in the mouse hippocampus [27]. Previous studies have used in situ hybridization to detect with high sensitivity the expression of lentivirus-delivered transgenes in the DG [12]. Therefore, we decided to use this technique to assess EGFP expression levels in the DG upon CMV-EGFP lentivirus delivery. As shown in Fig. 6A, in agreement with our previous observations on protein expression using EGFP native fluorescence, one week after injection the EGFP in situ hybridization signal was mostly restricted to the hilus and the SGZ, demonstrating that the lack of EGP expression in the outer layers of the GCL is not a consequence of possible post-transcriptional regulation but more likely of lack of transgene expression.

To substantiate this conclusion we should be able to show that there are no major physical obstacles to reach the DG by stereotaxic injection into the SR. To achieve this goal we used a fluorescently labeled transferrin-derived peptide (T12-Cy5, Prosensa BV, Leiden, The Netherlands) delivered by stereotaxic injection into the SR (1 μl; 30 μM). Transferrin-derived peptides have been shown to increase delivery efficiency of molecular cargos to neuronal cells in vivo [28,29]. Following this approach, 48 h after injection we observed fluorescence distributed across the SR and ML fields, reaching the CA1 and DG (Fig. 6B). A closer observation of the DG clearly displayed a fluorescence pattern with maximal intensity in the ML and gradually diffusing into the GCL, labeling Neuron-Specific Nuclear Protein (NeuN)+ cells present in all sub-layers of both blades of the GCL (Fig. 6C). These results demonstrated that our stereotaxic injections to the SR can positively transduce neurons of the GCL, including those located on the GCL3 and therefore that no physical (anatomical) obstacles may preclude lentivirus transduction of granule cells.

Although these findings support the conclusion that our lentiviral system is not able to induce transgene expression in GCL3 neurons, we tested once more this hypothesis in organotypic hippocampal slice cultures. Using this model, hippocampal cells are directly exposed to the virus-containing solution, avoiding the need of stereotaxic injection [19]. During the first postnatal weeks neurons of embryonic origin are already present in the immature GCL, while progenitor cells that will eventually complete the neuronal layer are still present in the hilus [30]. Four days after transduction with CMV-EGFP lentivirus the vast majority of the EGFP+ cells were negative for NeuN (Fig. 6D), therefore substantiating our conclusions. This is in agreement with previously reported observations, where we demonstrated that the CMV-EGFP lentivirus transduces nestin/GFAP+ neuronal progenitor cells in early postnatal hippocampal slices [19]. Moreover, others have shown that although transgene expression increases slowly with time after transduction of hippocampal slices with VSV-G pseudotyped lentivirus, it may remain restricted to CA1 and CA3 pyramidal cells [31].

**Figure 7:**
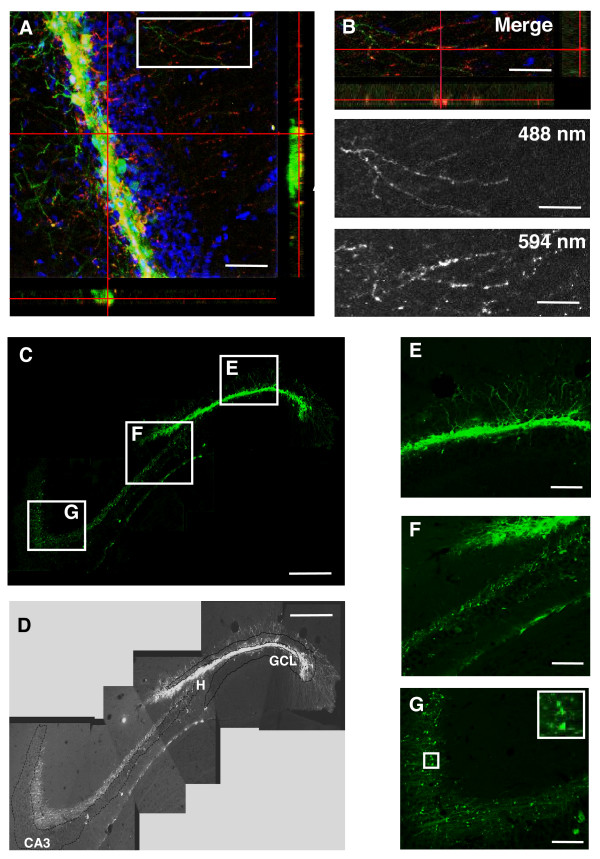
**Transduction pattern of the CMV-EGFP lentivirus in DG and CA3 by EGFP immunohistochemistry**. A: Confocal microscope image and orthogonal projections onto the x-z (bottom) and y-z (right) planes showing co-localization (yellow) of the native EGFP fluorescence (green) and EGFP immunohistochemistry signal (red) in GC somata, one week after injection. B: Higher magnification of the boxed area depicted in A showing partial co-localization in dendrites of GC (top). The split panels corresponding to the EGFP native fluorescence signal (488 nm, center) and the EGFP immunohistochemistry signal (594 nm, bottom) are shown. C and D: Composite of 5 confocal z-projected stacks combining EGFP's native fluorescence and immunohistochemistry signal 5 weeks after injection, showing EGFP positive cells with their somata in the GCL (E) and projecting axons into the hilus (F) and the Stratum Lucidum of the CA3 field (G), were the synaptic boutons of these axons are evident (F and inset in G). Each image shown is representative of 5 animals independently injected. Scale bars: A: 40 μm; B: 25 μm; C: 200 μm; E; F and G: 10 μm. GCL: *Granule Cell Layer*, H:*Hilus*. CA3: *Cornu Ammonis *3.

Since we have used EGFP native fluorescence to directly detect transgene expression, one possible technical limitation in observing positive cells could have been a presumable low sensitivity of native EGFP fluorescence detection. Indeed, in the reference protocol for transgene delivery to granule cells the use of EGFP immunohistochemistry and subsequent indirect fluorescence detection is recommended [7]. Therefore, we followed this approach to account for possible low detectability of EGFP-expressing cells. As shown in Fig. 7A, immunohistochemistry increased the detection of EGFP, as expected. Importantly, this increase was most evident in the dendritic arborizations and axonal extensions of the labeled cells. To account for this observation we used an Alexa 594-labelled secondary antibody to discriminate in between EGFP native fluorescence detected with an excitation wavelength of 488 nm and the immunohistochemistry signal detected with an excitation wavelength of 594 nm (Fig. 7A). This approach showed a partial colocalization of the two signals, with highest colocalization detected in cell somata, and only partial colocalization in dendrites and axons (Fig. 7A, boxed area and 7B). This phenomenon has been previously described and explained by partial antibody penetration under experimental conditions similar to ours [32]. Therefore, in successive experiments we utilized a combination of native fluorescence and immunohistochemistry signal by using an Alexa 488-labelled secondary antibody. Following this approach, 5 weeks after injection the morphology of EGFP expressing cells transduced by the CMV-EGFP lentivirus was exposed with great detail. As recently described with other retroviruses [32,33], we were able to observe the axonal projections of EGFP labeled granule cells into the hilus and the stratum lucidum of the CA3 (Fig. 7C–F). However, the distribution of EGFP+ cell somata within the GCL remained very similar to that observed using native fluorescence only, with almost no EGFP+ cells observed in the GCL3 (Fig. 7G). Altogether these experiments further substantiate our conclusion that cells within the GCL3 are less permissive to lentivirus-delivered transgene expression.

### Identity of EGFP+ cells in the GCL after CMV-EGFP injection

**Figure 8:**
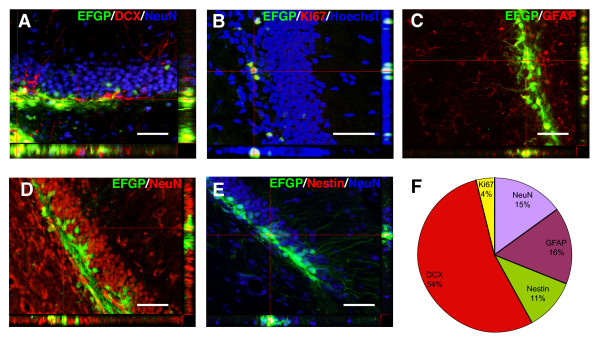
**Identification and quantification of different cell types targeted by injection of CMV-EGFP in the DG**. Examples of EGFP co-localization with different markers of neuronal differentiation within the GCL. The orthogonal projections onto the x-z (bottom) and y-z (right) planes of cells indicated by hairlines are shown to confirm double labeling throughout the extent of EGFP+ cells co-expressing DCX and NeuN (A); Ki67 (B); GFAP (C); NeuN (D) or Nestin and NeuN (E). Each image shown is representative of 5 animals independently injected. Scale bars: 20 μm. F: Percentual distribution of EGFP+ cells expressing differentiation markers within the GCL, 1 week after the stereotaxic injection normalized to the total number of EGFP+ cells.

**Figure 9:**
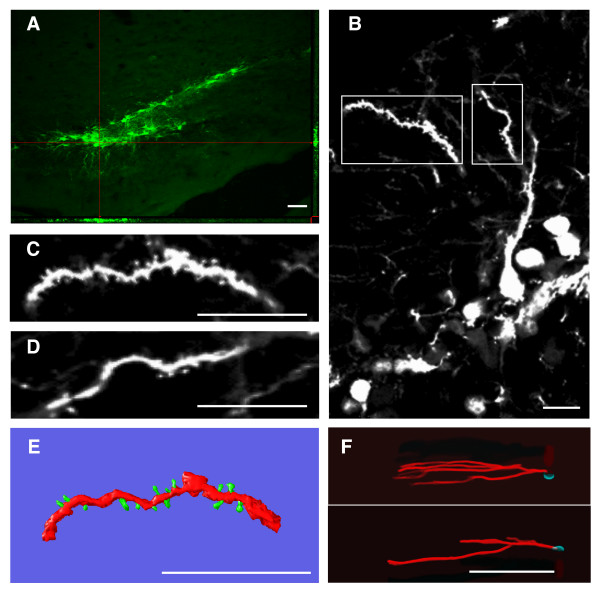
**Morphological analyses and three-dimensional reconstructions of EGFP+/NeuN+ cells in the GCL**. Representative examples of EGFP+ neurons located in the GCL 1 week after the stereotaxic injection of CMV-EGFP. (A) Confocal image showing EGFP+ cells in the DG. The orthogonal projections onto the x-z (bottom) and y-z (right) planes are shown to confirm EGFP expression throughout the extent of the cells indicated with hairlines. (B) Z-axis projection of EGFP+ neurons from the area depicted in A, showing their morphological features. C and D: higher magnification of the areas boxed in B. E: three-dimensional reconstruction of the dendritic segment depicted in C, shown as example of those used for dendritic protrusion analyses. F: Three-dimensional reconstructions of two example EGFP+ GCL neurons, showing their dentritic arborization and length. Cell somata are shown in cyan and dendrites in red. Similar neurons were used for quantitative analyses. Scale bars: A: 50 μm; B, C, D, E: 10 μm; F: 100 μm. (Full-resolution animated 3D-reconstructions are available at ).

To characterize the cell type(s) transduced by the CMV-EGFP lentivirus more accurately, we performed a series of immunohistochemical co-stainings for neuronal progenitor (nestin), glial (GFAP), immature neuron (DCX), proliferating (Ki67) and mature neuron (NeuN) cell markers [20] (Fig. 8). EGFP+ cells present in the GCL were analyzed for co-expression of these markers one week after lentivirus injection (Fig. 8A–E). Quantitative analyses of these samples demonstrated that the majority of the EGFP+ cells were DCX+, with phenotypes ranging from putative dividing neuronal progenitors to early post-mitotic immature neurons (Fig. 8). Nestin+, GFAP+ and NeuN+ cells accounted each for approximately one third of the EGFP+ cells, while Ki67 was coexpressed in a small proportion of the cells (Fig. 8F). NeuN+ cells were further analyzed for neuronal features such as the presence of dendritic spines (Fig. 9). We found that 11 ± 4% of the EGFP+ neurons present in the GCL had simple dendritic arbors with dendritic spines (Fig. 9B–D), phenotypicaly resembling immature, most probably adult generated neurons [8,24]. Quantitative analysis of spine density from EGFP+ neurons showed that these cells have relative low protrusion densities (Fig. 9E; 7 ± 2 protrusion/10 μm, n = 5 neurons, 420 protrusions counted) and present morphological features compatible with immature neurons [34]. Thee-dimensional reconstructions of EGFP+ cells revealed that these cells had narrow, low-complexity dendritic arbors, normally with one primary dendrite and relatively short secondary dendrites projecting into the ML (Fig. 9F; mean maximal distance from soma 203 ± 20 μm, n = 25 neurons), characteristics all compatible with being immature neurons [22,24].

## Discussion

In the present study we have used lentiviral vectors expressing EGFP from three different promoters in the mouse hippocampus and have identified sub-field specific differences in transgene expression in various cell types of the GCL of the DG. Furthermore, we have characterized the cell types transduced by these lentiviral vectors, concluding that they target primarily NPC and immature neurons present in the SGZ and more immature layers of the GCL. Our observations suggest the existence of intrinsic differences in the permissiveness to lentivirus transduction among populations of granule cells of the GCL. In particular, we show for the first time that mature neurons of the outer granule cell layer do not express lentivirus-delivered transgenes, despite successful expression in other hippocampal cell types. Therefore, only adult-generated neurons may be target for lentivirus-mediated transgene delivery within the GCL.

The DG of the mammalian hippocampus is progressively constructed through a complex developmental program. Embryology studies have demonstrated that the GCL can be divided into an outer shell and an inner core, originated from separate embryonic progenitor pools. These progenitors generate first the outer shell followed by the development of the inner core by later-born granule cells [35]. Therefore, the outer shell of the GCL is partially assembled during embryogenesis and the majority of dentate granule cells, located in the inner shell are generated after birth [36-38]. These and other observations have generated the hypothesis that, in contrast to the neocortex, the DG is built up following a life-long outside-in arrangement, where new cells are incorporated in the GCL following a downward gradient of positional cues [8].

In rodents, proliferative cells become largely confined to the SGZ at the base of the GCL after postnatal day 30 [37]. Therefore, during the juvenile and adult periods the SGZ is the source of newly produced granule cells [20].

Several groups have shown heterogeneous functional properties of granule cells in the adult hippocampus. In particular, new neurons generated by adult neurogenesis display increased synaptic plasticity and increased excitability suggesting that maturation of the neuronal phenotype includes changes in membrane excitability and morphology, as well as the establishment of appropriate connectivity [24,39,40]. Interestingly, it has been proposed that functional and morphological differences among granule cells are a function of their location within the GCL rather than of their relative age [23,41].

Herein we report that the three different lentivirus systems tested in this study, transduced mainly cells located in the SGZ and inner layers of the GCL. Cells expressing the reporter transgene EGFP one week after viral injection were mainly immature neurons expressing DCX. These observations resemble the EGFP expression profile achieved using MMLV-derived vectors that transduce only proliferating cells [24]. Therefore, the initial cell population hit by the lentivirus was most probably a subpopulation of NPC that evolved into the neuronal lineage as judged by the predominance of DCX+ cells one week after transduction, similar to reports using MMLV-vectors [22,24]. Moreover, retro- and lenti-viral vectors have been shown to target similar, although not completely overlapping, populations in the hippocampus [8]. Therefore, the use of adeno-associated virus-derived vectors may be more adequate to target mature neurons of embryonic origin in the adult dentate gyrus [42].

An indubitable characterization of the cell type originally transduced by the lentiviral vector may request the use of cell type specific promoters restricted to NPCs [43]. However, in the adult dentate gyrus, DCX is only expressed in cells contributing to adult neurogenesis and therefore can be used as a bona fide marker of newborn adult-generated neurons [44,45].

Our observations are in agreement with the described ability of lentiviral vectors to transduce adult NPC in vivo [46]. The presence of subpopulations of EGFP+ cells expressing the NPC marker nestin and Ki67, a cell proliferation marker expressed during the active phases of the cell cycle [47] emphasize our conclusions.

Moreover, the reduced numbers of EGFP+/NeuN+ cells found, their morphology and their location in the inner layers of the GCL, indicate that these EGFP+/NeuN+ cells have most probably originated from a population of immature cells originally hit by the virus.

Crucial to sustain these conclusions are our experiments in which we delivered the lentiviral vector to the SR, situated between the CA1 and the outer shell of the GCL. If the pattern of EGFP expression restricted to the inner layers of the GCL would have been a mere mechanical effect of the steric hindrance generated by the tightly packed structure of the GCL [48], the lentiviral vector should have been able to transduce cells in the outer layers of the GCL, when delivered to the SR. Conversely, we observed strong EGFP expression in cells within the ML and CA1, demonstrating adequate diffusion of the lentivirus across different cellular structures. Moreover, EGFP+ cells were homogenously distributed within the CA1 layer, with profuse EGFP expression in the soma, axons and dendrites of cells phenotypicaly resembling mature pyramidal neurons. Our experiments using a peptide-cy5 conjugate, depicted in Fig. 6, showed that this construct delivered into the SR, could effectively transduce the neurons located in the outer layers of the suprapyramidal blade of the GCL and beyond into the hilus and the infrapyramidal blade. These experiments demonstrated that stereotaxic injection to the SR permits effective delivery to the GCL.

Our data from the CA1 cells demonstrated as well that the CMV promoter is indeed able to promote transgene expression in mature postmitotic neurons, as previously described [26]. These observations made us to conclude that, although the use of different (cell-type specific) promoters is useful to promote different patterns of transgene expression in the GCL, cells present in the outer shell of the GCL only scarcely express transgenes delivered by lentiviral vectors. Interestingly, the Synapsin I promoter rendered an EGFP expression profile more similar to that of the CMV promoter than to that of the CaMKII promoter, in accordance to its expression in earlier neuronal developmental stages [10]. Therefore, although further experiments to investigate transgene expression mediated by different promoters at later times post-injection seems important to address the relevance of differential promoter use, it escapes the objective of the present study.

One potential drawback of the use of the CMV promoter may be its potential activation in astrocytes short time after injury, described in the cerebral cortex and caudate-putamen [49]. Nevertheless, this activation could be dependent on virus titers and other factors such as the particular CMV sequence used and the time after the injection [50]. For the interpretation of the data presented herein it is worth to take into account that sections surrounding the injection site were routinely discarded.

Specific transgene silencing after lentiviral vector-mediated delivery has been described before [51]. Although we can not exclude from this set of experiments the possibility that transgene expression driven by the three promoters used in this study were selectively silenced in mature neurons present in the outer layers of the GCL, the fact that the CMV promoter was able to promote expression in cells of the CA1 makes this possibility unlikely.

Overall, our observations are in agreement with previous reports showing that lentiviral vectors can successfully transduce mitotic and postmitotic cells [26,46,52]. However, the exact nature of the cell types and hippocampal sub-fields targeted by lentiviral vectors remains controversial. Previous reports did not find sub-field specific differences in GFP expression. This could be due to technical differences such as the use of different GFP variants and constructs, analysis of the samples at different time points after stereotaxic injection or differences in the CMV promoter sequence used to control transgene expression [6,12]. Nevertheless, the disparity in EGFP expression reported herein between cells located in the inner or outer layers of the GCL seems to be a function of intrinsic differences between cells generated by embryonic or adult neurogenesis. In this context, disparities in transgene expression in granule cells, depending on their relative location within the GCL and their progression into the neuronal differentiation program, emphasize the heterogeneity between newly adult-generated neurons and pre-existing ones, probably originated during embryonic and/or early postnatal development.

Although further experiments will be required to clarify the exact nature of this heterogeneity among granule cells of the DG, regarding their permissiveness to lentivirus-delivered transgene expression, one possible explanation could be the differential expression of receptor proteins that recognize pseudotyping proteins by subpopulations of granule cells. However, VSV-G pseudotyped viruses have been shown to effectively transduce cells within the GCL of the DG [6,12]. This suggests that, although pseudotyping proteins can influence transduction efficiency and tropism to hippocampal cell types [11,25], the receptors for VSV-G glycoprotein are present in granule cells of the DG. Moreover, transgene expression from VSV-G pseudotyped lentivirus is pantropic in the rat brain, labelling a variety of glial and neuronal cell types depending on the promoter used to control transgene expression [52].

Interestingly, even though cell mitosis is not a requisite for integration, transduction efficiency of lentiviral vectors is dependent on cell-cycle progression of target cells, with cells actively growing or arrested in phases other than G_0 _being more efficiently transduced in vivo [26,53-55]. As demonstrated here, lentivirus transduced EGFP+ cells are in their vast majority positive for progenitor (nestin), astrocyte (GFAP), proliferation (Ki67) and immature neuron (DCX) cell markers. Furthermore, Schmetsdorf et al [56] have demonstrated that cells from distinct hippocampal fields, including CA1, CA3 and DG, express completely different repertoires of cell cycle-related proteins. Therefore, although a more thorough elucidation of the factors regulating lentivirus transduction of postmitotic granule cells is beyond the scope of this article, our observations demonstrating lentivirus-mediated transgene expression in NPC and immature neurons suggest that cell-cycle progression is an important determinant in lentivirus transduction efficiency of hippocampal granule cells in vivo.

## Conclusion

Herein, we report on sub-field specific differences in permissiveness to lentivirus-delivered transgene expression in the mouse hippocampus. Most interestingly, we observed transgene expression preferentially in NPC and immature neurons present in the SGZ and inner layers of the GCL, where adult neurogenesis takes place and different subpopulations of granule cells exist. Based on our results, we conclude that this disparity in transgene expression observed between cells located in the inner or outer layers of the GCL seems to be a function of intrinsic differences between cells generated by embryonic or adult neurogenesis and therefore favour the hypothesis that cell-cycle progression of target cells is an important determinant of lentivirus transduction efficiency. These differences could be exploited in utilizing lentivirus for transgene delivery to NPC and immature neurons of the mouse hippocampus in vivo.

## Methods

### Experimental setup

We investigated expression of EGFP and cell-type specific markers in hippocampal cells after transduction with AGLV [57]. In these vectors, EGFP expression was under the control of three different Polymerase II promoters, as described in the Results section [10,19]. Hereto, animals were divided into experimental groups of 5 animals each and intra-hippocampally injected into the DG or SR with one of the three types of lentivirus. One or five weeks after injection, brain tissue was processed for immunohistochemistry.

### Cloning and Lentiviral vector production

Replication incompetent and self-inactivating Advanced Generation lentiviral vectors were produced and titrated as previously described [19]. All lentivirus batches used for experiments had comparable titers ranging from 1 × 10^8 ^to 1 × 10^9 ^transducing U/ml. Virus suspensions were stored at -80°C until use and were briefly centrifuged and kept on ice immediately before injection.

### Animals

Male C57Bl/6J mice (seven weeks old at injection, Janvier Biosciences, France) were housed 5/cage for one week before surgery as acclimatization. Thereafter, mice were single housed in filtertop cages, in a temperature and humidity controlled room with 12:12 dark-light cycle (light on at 08:00 A.M.). Mice had free access to food pellets and water. All efforts were made to minimize animal suffering and the number of animals used. All experiments were approved by the committee of Animal Health and Care, Leiden University and The Netherlands Commission for the Use of Genetically Modified Organisms and performed in compliance with the European Union recommendations for the care and use of laboratory animals.

### Stereotaxic surgery

Stereotaxic injections were performed essentially following previously described methods [7]. Animals were deeply anaesthetized by a mixture of Hypnorm (0.5 mg/kg/ml) and Dormicum (5 mg/kg/ml) and Milli-Q purified water (Millipore, Amsterdam, The Netherlands) at volume ratio of 1:1:2 (10 ul/g). Bilateral injections of lentiviral vectors into the Dentate Gyrus (AP: -2.00 mm, ML: +/-1.50 mm, DV: -1.90 mm, relative to Bregma) or the Stratum Radiatum (AP: -2.00 mm, ML: +/-1.50 mm, DV: -1.50 mm, relative to Bregma), were conducted using a small animal stereotact (900 series, David Kopf Instruments, Tujunga, CA) and an injection pump (Harvard Apparatus, Holliston, MA) with injection volume = 1 μl, rate = 0.4 μl/min, connected to a Hamilton needle (5 μl, 30 gauche), and customized borosilicate glass micro-capillar tips of approximately 100 μm. After surgery animals were placed under a heating lamp until awakening and further monitored and weighted daily.

### Immunohistochemistry

One or five weeks after injection, animals were sacrificed and brains were fixed by transcardial perfusion. Before the procedure the animals were deeply anaesthetized by IP injection of sodium pentobarbital (Nembutal 60 mg/ml, 0.1 ml). Animals were transcardialy perfused with 0.1 M PBS for 10 minutes. Brains were removed and kept in 25 ml 4% PFA for one hour. Then, they were washed in 0.1 M PBS and immersed in 15% and subsequently 30% sucrose solution for 3–4 days. Brains were blotted dry and snap-frozen for 10 sec in isopentane on dry ice and stored at -80°C until sectioning.

Serial coronal 20 μm-thick sections, were obtained using a cryostat (Leica CM 1900, Leica Microsystems, Rijswijk, The Netherlands). All brain sections containing the hippocampus were collected and thaw-mounted on SuperFrost microscope slides and stored at -80°C until further use.

Immunofluorescent double and triple labelling was performed as described [58]. Primary antibody were from: Santa Cruz Biotechnology, Inc; Heidelberg, Germany (Doublecortin (C-18), used 1:200; Ki67 (M-19), used 1:100; GFAP, mouse monoclonal, used 1:1000); Chemicon-Millipore International BV, Amsterdam, The Netherlands (NeuN (A60), used 1:200), BD Biosciences, Breda, The Netherlands (Nestin, (556309), used 1:200) or Molecular Probes/Invitrogen, Breda, The Netherlands (GFP, chicken polyclonal, used 1:500). After 24 h incubation at 4°C with continuous stirring, sections were incubated with correspondent Alexa488 or Alexa594-conjugated secondary antibodies (1:400, Molecular Probes/Invitrogen) for 2 hrs at RT in 100 μl 1 × PBS/0.3% TritonX-100. Sections were counterstained with Hoechst 33342 when indicated, as previously described [58]. Sections were embedded with Aqua-Poly/Mount (Polysciences Europe, Eppelheim, Baden-Württemberg, Germany). Similar samples were processed in parallel excluding primary antibodies and used for comparison as negative controls (not shown).

### Organotypic hippocampal slice cultures

Early postnatal rat hippocampal slices were produced an cultured as previously described [19]. Briefly, slice cultures were prepared from 4- to 6-day-old male Wistar rats (Charles River Laboratories, Inc., Frankfurt, Germany) using the modified interface culture method. At the time of the first medium change (day in vitro (DIV) 1), hippocampal slices were inoculated with 10 μl of the CMV-EGFP lentiviral vector stock. Slices were fixed 4 days later with 4% paraformaldehyde for 1 h at 4°C and used for immunofluorescence studies.

### In situ hybridization for EGFP mRNA

Perfused mouse brain sections were used for in situ hybridization with a 720 basepair long digoxigenin (DIG)-labeled EGFP riboprobe (antisense to NCBI gene ID DQ768212). The in situ hybridization was performed essentially as described by Schaeren-Wiemers and Gerfin-Moser [59], with small modifications. Briefly, sections were fixed in 4% paraformaldehyde (PFA) for 5 minutes, treated for 10 minutes with 10 ug/ml proteinase K and 0.1% Triton-X100 in phosphate buffered saline (PBS, pH 7.4), followed by 10 minutes extra fixation with 4% PFA. Thereafter, sections were rinsed 3 times in PBS for 3 minutes. After acetylation for 10 minutes (0.25% acetic anhydride in 0.1 M triethanolamine), sections were washed 3 times in PBS for 5 minutes and prehybridized for 2 hours at room temperature in hybridization solution, containing 50% deionized formamide, 5× SSC, 5× Denhardt's solution, 250 μg/ml tRNA Baker's yeast and 500 μg/ml sonicated salmon sperm DNA. 150 μl of hybridization mixture containing 400 ng/ml DIG-labeled riboprobe was applied per slide, covered with Nescofilm and hybridized overnight at 68°C. The next morning slides were quickly washed in 2× SSC followed by 0.2× SSC for 2 hours, both at 68°C. DIG was detected with an alkaline phosphatase labeled antibody (1:5000, Roche, Mannheim) using NBT/BCIP as a substrate. After DIG in situ hybridization, slides were counterstained with 0.5% methyl green, quickly dehydrated in ethanol, cleared in xylene and mounted using Entellan.

### Histological analyses and confocal microscopy

Quantification of EGFP+ cells and quantitative analysis of different classes of neuronal cells in the hippocampus of treated animals were performed using the optical fractionator sampling method, as described by Encinas and Enikolopov [60]. Briefly, every tenth hippocampal section was collected starting at the DG following the fractionator scheme, to ensure that each slice is 200 nm apart from the next slice within each collected set of approximately 11 slices [60]. For quantification of EGFP+ cells, three sets of slices from at least three independently injected animals from each experimental group were used. Sections surrounding the injection site were routinely discarded. For quantitative analysis of neuronal cell-types other three sets of slices from at least three independently injected animals from each experimental group were used. Confocal images were acquired using a Nikon C1si Spectral confocal microscope, as described [19]. Expression of markers and cell-localization analyses were done counting more than 50 EGFP+ cells per animal. Co-localization was assessed through the entire z-axis of each cell, using an optical slice of 0.3–0.6 μm. Morphology was analyzed from three-dimensional reconstructions of series of sequential confocal images taken at 0.3–0.6 μm intervals in EGFP+ cells.

### Image analysis

For EGFP+ cell-localization analyses within the DG or CA1 sub-fields, maximum intensity z-axis projections of series of sequential confocal images were constructed using ImageJ, as described [19]. Using these projections, EGFP+ cells were automatically identified and counted using Cell Profiler [61]. This procedure was validated by comparison to manual counting performed by an experienced operator using the optical fractionator method sampling scheme and unbiased stereology estimation of cell numbers as described by West and co-workers [62]. The "pipeline" used to automate cell counting was composed of the following Cell Profiler's modules, in the specified order: LoadSingleImage, ColorToGray, CorrectIllumination_Calculate, CorrectIllumination_Apply, IdentifyPrimAutomatic. By using this pipeline we routinely found a strong correlation between the manual unbiased stereology method and the automated procedure (r = 0.985, Pearson's correlation test performed with GraphPad Prism 4, GraphPad Software, Inc., La Jolla, CA). EGFP+ cells were individually pseudo-colored to facilitated visualization and cell-localization maps were generated using Cell Profiler. Subsequently, based on a previously described manual method to study granule cell location within the GCL [63] the GCL was subdivided in four 2-cell-body-wide sub-layers using ImageJ  to generate a superimposed grid, guided by Hoechst 33342 staining of cell nucleus. These sub-layers were denominated: subgranular zone (SGZ) and granule cell layer (GCL) 1 to 3, as described by others [8,23,24]. Then, the pseudo-colored cell-localization maps generated with Cell Profiler were used to manually assign and count individual EGFP+ cells to the 4 sub-layers of the GCL of the DG. In all cases, EGFP+ cells present in the apex of the DG were excluded from the analyses. A similar procedure was used in experiments comprising EGFP+ cells in CA1.

For quantification of different cell-type markers in EGFP+ cells, total EGFP+ cells were automatically identified and counted using Cell Profiler from z-projected confocal images. From the same images, cells positive for each individual co-stained marker were also automatically identified and counted with Cell Profiler using the corresponding confocal channel. Cells positive for each marker analyzed were expressed as percentage of total EGFP+ cells. All image analyses procedures were performed in hippocampal slices from at least three independently injected animals as described above. In all cases, image analyses were performed by an operator blind to treatment.

### Dendrite tracing and three-dimensional reconstructions

Three-dimensional reconstructions of dendritic arbors and spine density analysis were performed using TDR3D software package , using a simulated fluorescence process-based algorithm [64,65]. Briefly, three-dimensional reconstructions for morphological analyses were generated from series of confocal images of EGFP+ neurons taken at 0.3–0.6 μm intervals from at least three independently injected animals. All cells used for morphological analyses were positive for the neuronal marker NeuN (not shown). Quantification of dendritic protrusions and dendritic lenght was done with ImageJ (NeuronJ plugin).

## Authors' contributions

LWAvH performed the stereotaxic injections, immunohistochemical and histological procedures, discussed the results and participated in the preparation of the manuscript, MI participated in the immunohistochemical and histological procedures and performed image analyses, TFD participated in the experimental design and discussed the results, TGS performed the cloning and lentiviral vector production, MdB and RAHA designed and performed the EGFP in situ hybridization experiments, FJV supervised and participated in the analyses of the three-dimensional reconstructions, EV conceived and supervised the experiments, discussed results, corrected the manuscript and provided financial support, CPF participated in the experimental design and image analyses, performed confocal microscopy experiments and three-dimensional reconstructions, supervised the experiments, discussed results and prepared the manuscript.
